# Characterization and protective efficacy of a *sptP* mutant of *Salmonella* Paratyphi A

**DOI:** 10.1002/iid3.369

**Published:** 2020-11-01

**Authors:** Pengtao Pan, Fanyu Zou, Chuanshan He, Qunli He, Junlei Yin

**Affiliations:** ^1^ Medical College Xinxiang University Xinxiang China; ^2^ Second Hospital Shanxi Medical University Taiyuan China; ^3^ Medical College Zhengzhou University of Industrial Technology Zhengzhou China

**Keywords:** paratyphoid fever, pathogenicity, *Salmonella* Paratyphi A, *sptP*, vaccine

## Abstract

**Background:**

*Salmonella* Paratyphi A causes paratyphoid A, a severe systemic disease of people and remains a major public health problem in many parts of the world. In the interest of researching the roles of *sptP* on *Salmonella* Paratyphi A and developing a live‐attenuated vaccine candidate, an *sptP* mutant of *Salmonella* Paratyphi A SPA017 (SPA017Δ*sptP*) was constructed, and then its characterization, immunogenicity, and protective ability were evaluated.

**Results:**

The deletion of *sptP* had no effect on growth and biochemical properties. Adhesion and invasion assays showed that the lack of *sptP* did not affect the adhesion of *Salmonella* Paratyphi A, but the invasive ability of the mutant strain was significantly decreased, the half‐lethal dose (LD_50_) of the mutant strain was 1.43 × 10^4^ times of the parent strain in intraperitoneally injected mice. Single intraperitoneal vaccination with SPA017Δ*sptP* (1 × 10^5^ CFU) in mice did not affect the body weight or elicit clinical symptoms relative to the control group, SPA017Δ*sptP* bacteria were isolated from livers and spleens of vaccinated mice at 14 days postvaccination. Notably, specific humoral and cellular immune responses were significantly induced. The protective assessment showed that the mutant strain could provide high‐level protection against subsequent challenge with the wild‐type SPA017 strain.

**Conclusions:**

These results demonstrated that SptP plays an essential role in the pathogenicity of *Salmonella* Paratyphi A, and *Salmonella* Paratyphi A lacking *sptP* is immunogenic and protective in mice.

## Graphical abstract

*Salmonella* Paratyphi A lacking sptP is attenuated, immunogenic, and protective in mice. The sptP mutant SPA017ΔsptP has the potential of being a live‐attenuated vaccine candidate.

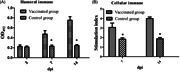

**Abbreviations**
LB: Luria‐Bertani
LD_50_: half‐lethal dose
SI: stimulation index
SPI1: *Salmonella* pathogenicity island 1
T3SS: type III secretion system

## 1 INTRODUCTION

Pathogenic bacteria belonging to the genus *Salmonella* can cause a series of diseases in humans and animals.^1^ *Salmonella* Paratyphi A is the pathogen of paratyphoid A which can cause typhoid‐like clinical symptoms characterized by prolonged fever, headache, loss of appetite, nausea/vomiting, abdominal pain, and diarrhea, which can be life‐threatening.^2^ People are generally susceptible to *Salmonella* Paratyphi A, but the incidence is highest in children and young adults, and in severe cases, death may occur.^3^, ^4^ Also, it has been reported that the bacteria of *Salmonella* Paratyphi A can transmit vertically and results in abortion.^5^ In recent years, the incidence of paratyphoid A has increased and remains high in many parts of the world, and has become a major public health problem.^2^, ^6^

Drug therapy is a standard option to deal with *Salmonella* Paratyphi A infection, but the increase of drug resistance and the emergence of multi‐drug resistance has become a thorny problem in the treatment of *Salmonella* Paratyphi A infection.^7^ Currently, there are no commercial vaccines specifically targeting *Salmonella* Paratyphi A infection in the market. Therefore, it is necessary to carry out the research and development of *Salmonella* Paratyphi A vaccine. Type III secretion system (T3SS), whose central function is to deliver the bacterial protein into host cells is a vital strategy evolved by *Salmonella* to infect host cells.^8^ *Salmonella* encodes two T3SSs (T3SS1 and T3SS2), they are encoded on *Salmonella* pathogenicity island 1 (SPI1) and SPI2, respectively. The function of T3SS1 and T3SS2 is different, T3SS1 can translocate a set of effectors to promote the invasion of *Salmonella* into host cells, then T3SS2 is induced and mediates systemic infection of *Salmonella* in the host.^9^ As a *Salmonella* protein tyrosine phosphatase, SptP encoded within SPI1 is one of the T3SS1 effectors, and plays essential roles in downregulating membrane ruffling and *Salmonella*‐containing vacuole maintenance.^10^ SptP is also a modular protein, including two distinct domains: (1) the N‐terminal domain, which acts as a GTPase‐activating protein for Rac1 and Cdc42. It mediates the reversion of the actin cytoskeleton changes; and (2) the C‐terminal half of SptP, which has a similar sequence with prokaryotic tyrosine phosphatase YopH of *Yersinia* and eukaryotic tyrosine phosphatases.^11^, ^12^ A chaperone, SicP, is required for the stability within the bacterial cytosol and secretion of SptP.^13^, ^14^ The virulence of *Salmonella enteritidis* was reduced considerably with the absence of *sptP*, the mutant could be applied as a vaccine to protect against *Salmonella enteritidis* infection.^15^ This provides an idea for the development of paratyphoid A vaccine based on the lack of *sptP*.

Herein, a *sptP* deletion mutant of *Salmonella* Paratyphi A was established. Subsequently, we assessed its features, including growth rate and biochemical properties. Thus, we conducted adhesion and invasion assays to examine its virulence. Furthermore, we evaluated the protective and immunogenicity of the mutant to develop a live‐attenuated vaccine candidate against *Salmonella* Paratyphi A.

## 2 MATERIALS AND METHODS

### 2.1 Bacterial strains, plasmids, and primers

The wild‐type *Salmonella* Paratyphi A strain SPA017 was stored in our laboratory,^16^ the *sptP* deletion mutant SPA017Δ*sptP* and SPA017Δ*sptP*::*cat* (chloramphenicol resistance, Cm^r^) were constructed using lambda Red recombination system with the primer *sptP*‐*cat*‐F/*sptP*‐*cat*‐R, and plasmids pKD3, pKD46, and pCP20,^17^ the complementation CoSPA017Δ*sptP* (ampicillin resistance, Amp^r^) was constructed with the plasmid of pBR322 through ClonExpress^TM^ II One Step Cloning Kit (Vazyme Biotech Co., Ltd). Bacteria were grown in Luria‐Bertani (LB) broth, LB agar (solidified with 1.5% (wt/vol) agar), or XLT4 (Difco) agar media according to different needs. The media were supplemented with Amp (100 μg ml^−1^)/Cm (34 μg ml^−1^) depending on the need. The primers used herein are listed in Table 1.

**Table 1:** Primers used in this study

Primers	Sequences (5′−3′)	Production size (bp)	Usage	Source
*sptP*‐*cat*‐F	TTGAGTCATTTGTGAATAAGCAGGAAGCGCTCAAAAACATACTACAGGAATtgtgtaggctggagctgcttcg	1115	Capital letter: *sptP* homologous arm; small letter: Cm^r^ cassette amplification	This study
*sptP*‐*cat*‐R	ACAGAAATAGCTTACTTTCAGATAGTTCTAAAAGTAAGCTATGTTTTTAcatatgaatatcctccttag			
*sptP*‐out‐F	CCATTGGTCATAACCGAGAT	2178 or 631	Identification of *sptP* mutant	This study
*sptP*‐out‐R	GGCTGCGAATAATGAAGGT			
*sptP*‐in‐F	TTGGTCTATCGCACCTCCC	269 or 0	Identification of *sptP* mutant and its complementation	This study
*sptP*‐in‐R	GAATGCCTGTGCCAGTGAA			
CoΔ*sptP*‐F	cagcttatcatcgat**aagctt**TCAGCTTGCCGTCGTCATAAGCA	1773	Small letter: homologous sequences of pBR322; capital letter: *sptP* cassette amplification; bold: restriction sites	This study
CoΔ*sptP*‐R	tgcgtccggcgtaga**ggatcc**TACATGCAATTACCGATCTGAC			

### 2.2 Growth and biochemical testing

The influence of *sptP* gene on the growth rate of *Salmonella* Paratyphi A was determined as previously described.^16^ Briefly, bacteria SPA017, SPA017Δ*sptP*, and CoSPA017Δ*sptP* were inoculated in 5 ml LB broth, then maintained in a shaker (180 rpm) overnight at 37°C. Subsequently, each culture was diluted to OD_600_ of 0.05 with LB broth, and this was used as the starting concentration (0 h). The culture was grown for 16 h, and the OD_600_ was determined every 2 h. Then the biochemical characteristics of SPA017, SPA017Δ*sptP*, and CoSPA017Δ*sptP* were performed using micro‐biochemical tubes (Qingdao Hopebio‐Technology Co., Ltd.) as described previously.^16^

### 2.3 Invasion and adhesion assays

We conducted the assays mentioned above using Caco‐2 BBE (human epithelial cells) and RAW264.7 (mouse macrophage cells), as previously described.^15^ In brief, cells (2 × 10^5^) from each cell line were seeded into 24‐well plates 24 h before infection, then each cell lines were infected with SPA017, SPA017Δ*sptP*, and CoSPA017Δ*sptP* for 30 min (RAW264.7) or 1 h (Caco‐2 BBE) at a multiplicity of infection of 100:1, respectively. For adhesion, the cells were rinsed three times and lysed in 1 ml of phosphate‐buffered saline (PBS) with 0.5% Triton X‐100 at 37°C for 10 min, for invasion, the plates were incubated for an additional 30 min in culture medium with 100 μg ml^−1^ gentamicin after adhesion and the cells were washed and lysed in PBS with 0.5% Triton X‐100 at 37°C for 10 min, then the bacteria number was counted. The results of adhesion and invasion were expressed as the fold change compared to the bacterial number of SPA017Δ*sptP*, respectively.

### 2.4 Virulence assessment

Female BALB/c mice (8‐weeks‐old) were obtained from the Experimental Animal Center of Xinxiang Medical University. The animal experiments were approved by the Animal Care and Ethics Committee of Xinxiang University, Xinxiang, China.

To determine the virulence of SPA017Δ*sptP* in BALB/c mice, 24 mice were divided randomly into four groups (*n* = 6), each group was inoculated intraperitoneally with 10X dilutions of SPA017Δ*sptP* from 1 × 10^8^ to 1 × 10^5^ CFU in 100 μl PBS; another 36 mice were injected intraperitoneally with SPA017 or CoSPA017Δ*sptP*; six control mice were given 100 μl of PBS in the same manner (Table 2). Records of deaths were taken over 14 days. The Karber and Behrens method was applied in the calculation of the half‐lethal dose (LD_50_) as described previously.^18^

**Table 2:** LD_50_ of SPA017, SPA017Δ*sptP*, and CoSPA017Δ*sptP* in mice after intraperitoneal injection

Strains	Challenge dose (CFU)	No. of deaths/total no. of mice	LD_50_ (CFU)
SPA017	1 × 10^3^	6/6	1.00 × 10^2^
	1 × 10^2^	3/6	
	1 × 10^1^	0/6	
SPA017Δ*sptP*	1 × 10^8^	6/6	1.43 × 10^6^
	1 × 10^7^	5/6	
	1 × 10^6^	3/6	
	1 × 10^5^	0/6	
CoSPA017Δ*sptP*	1 × 10^3^	6/6	1.00 × 10^2^
	1 × 10^2^	3/6	
	1 × 10^1^	0/6	
PBS	–	0/6	–

Abbreviations: CFU, colony‐forming unit; LD_50_, half‐lethal dose; PBS, phosphate‐buffered saline.

### 2.5 Changes in body weight and clinical symptoms

To assess the effects of the mutant SPA017ΔsptP on the growth of the mice, twenty‐seven 8‐weeks‐old female BALB/c mice were divided randomly into Group 1 (*n* = 11, received 2 × 10^5^ CFU of SPA017Δ*sptP* in 100 μl PBS intraperitoneally) and Group 2 (*n* = 16, received 100 μl PBS). We measured the body weights of the mice at 3, 7, and 14 days postinoculation (dpi). Clinical symptoms (such as diarrhea, anorexia, depression, mortality, and morbidity) were also observed, and daily records taken from Day 1 to 14 postinoculation.

### 2.6 Colonization and persistence assay

To assess the persistence of the mutant SPA017Δ*sptP* in immunized mice, three mice from per group were euthanized at 7 and 14 dpi, liver and spleen samples were aseptically collected for bacterial recovery. The weight of the samples was weighed, and PBS was added to make a suspension. One‐hundred microliters of homogenates of different dilutions were inoculated on XLT4 agar counted after 20 h at 37°C. The result was expressed as log10 CFU/g, and the negative sample was considered as 0 CFU/g.

### 2.7 SPA017Δ*sptP*‐mediated immune responses

At 3, 7, and 14 dpi, the serum immunoglobulin G (IgG) levels were determined using heat‐inactivated *Salmonella* Paratyphi A SPA017 as a coating antigen.^16^ Blood samples were collected from mouse tail veins, and then serum samples were separated, serum samples (diluted 1:50) and horseradish peroxidase‐conjugated goat anti‐mouse IgG (diluted 1:10,000) were used as the primary antibody and the secondary antibody, respectively. The result was shown using absorbance at 492 nm using an ELISA reader.

The cellular immune responses were determined by peripheral mononuclear cell proliferation assay at 7 and 14 dpi.^19^, ^20^ The wild‐type *Salmonella* Paratyphi A strain SPA017 was used to prepare a soluble antigen. Peripheral lymphocytes were separated from three blood samples per group at each time point. After Trypan blue dye exclusion testing, each well of a 96‐well plate was inoculated with 100 μl of a lymphocyte suspension at a concentration of 5 × 10^6^ cells/ml suspended in Roswell Park Memorial Institute 1640 medium (supplemented with 10% fetal calf serum, 50 U/ml penicillin, 50 μg/ml streptomycin, and 2 mM l‐glutamine) for 48 h at 37°C in a humidified 5% CO_2_ atmosphere. Lymphocyte proliferation activity was measured by adenosine triphosphate bioluminescence, and the result was expressed by the stimulation index (SI).^20^

### 2.8 Protection assessment

At 14 dpi, five animals were randomly taken from Group 1 and named Group A, and 10 mice were randomly taken from Group 2 and divided into Groups B and C with five animals in each group. Group A and B were subjected to intraperitoneal injection of *Salmonella* Paratyphi A strain SPA017 at a dose of 1 × 10^3^ CFU per mouse. Group C was inoculated with 100 μl PBS per mouse. Subsequently, daily records of surviving animals and clinical symptoms, such as anorexia, depression, diarrhea, mortality, and morbidity were taken daily for 15 days postchallenge.

### 2.9 Data analysis

Data are presented as the mean ± *SEM*. All the analyses were performed using GraphPad Prism. *p < .05 and **p < .01 represented two levels of statistical significance when using a one‐way analysis of variance.

## 3 RESULTS

### 3.1 Growth and biochemical characteristics of the mutant SPA017Δ*sptP*

**Figure 1:**
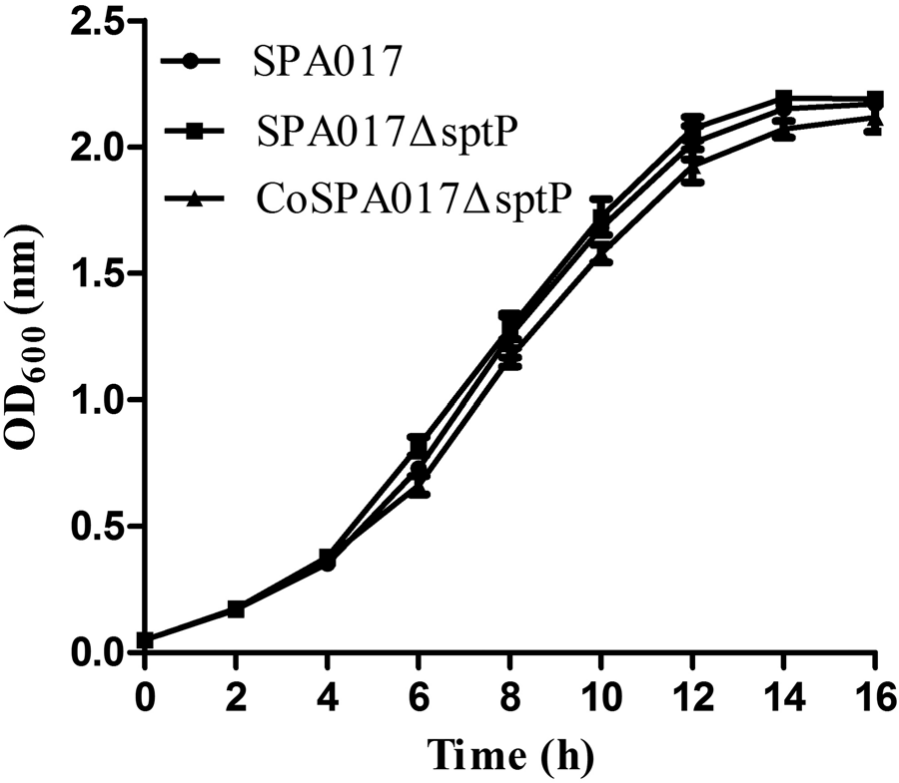
Growth curves of the parental strain SPA017, the mutant strain SPA017Δ*sptP* and the complementation strain CoSPA017Δ*sptP* in LB broth. All strains showed an identical growth response. Values represent the mean ± *SEM*. LB, Luria‐Bertani; OD, optical density

Growth curve analysis revealed that the wild‐type strain SPA017, the mutant SPA017Δ*sptP*, and the complementation CoSPA017Δ*sptP* grew at a very similar rate in LB broth at 37°C (Figure 1).

The biochemical tests showed that the mutant SPA017Δ*sptP* were positive in arabinose, mannitol, glucose, methyl‐red, rhamnose, and ornithine decarboxylase, and negative in citrate, glycerol, H_2_S, indole, lysine decarboxylase, lactose, oxidase, sucrose, phenylalanine, urea, xylose, and Voges–Proskauer in the same fashion to the wild‐type strain SPA017 and the complementation CoSPA017Δ*sptP*.

### 3.2 Adhesion and invasion analysis in cells

**Figure 2:**
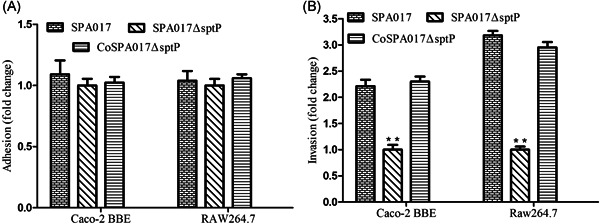
Bacterial adhesion (A) and invasion (B) of the parental strain SPA017, the mutant strain SPA017Δ*sptP* and the complementation strain CoSPA017Δ*sptP* in human epithelial Caco‐2 BBE cells and mouse macrophage RAW264.7. The result is expressed as the fold change compared to the bacterial number of SPA017Δ*sptP*. Values represent the mean ± *SEM*. **p < .01

Adhesion and invasion assays were performed in RAW264.7 and Caco‐2 BBE. The deletion of *sptP* gene did not affect the ability of adhesion of *Salmonella* Paratyphi A in cells of RAW264.7 and Caco‐2 BBE. However, the ability of invasion of the mutant SPA017Δ*sptP* is significantly lower than that of SPA017 and CoSPA017Δ*sptP* (Figure 2), implying the deletion of *sptP* gene from *Salmonella* Paratyphi A could decrease the ability of invasion (*p* < .01) significantly.

### 3.3 Determination of LD_50_

The LD_50_ of SPA017, SPA017Δ*sptP*, and CoSPA017Δ*sptP* were 1.00 × 10^2^ CFU, 1.43 × 10^6^ CFU, and 1.00 × 10^2^ CFU, respectively (Table 2). The LD_50_ of the mutant SPA017Δ*sptP* was 1.43 × 10^4^ times higher compared to that of the wild‐type strain SPA017, indicating that the mutant virulence reduced remarkably in comparison to the wild‐type strain.

### 3.4 Effect of vaccination on body weight and associated clinical symptoms

No significant difference was observed regarding the mean body weight of mice in Group 1 (immunized with SPA017Δ*sptP*) and Group 2 (mock group) at 3, 7, and 14 dpi (Table 3). Observation of the clinical symptoms showed that the immunized mice were in a normal state relative to the control group, without symptoms of diarrhea, anorexia, depression, mortality, and morbidity.

**Table 3:** Mean body weights of mice after vaccination

Group	Mean body weight per mouse at dpi (g)		
	3	7	14
1	21.554 ± 0.473	22.382 ± 0.664	22.936 ± 0.516
2	20.848 ± 0.716	21.597 ± 0.690	22.988 ± 0.847

Abbreviation: dpi, days postinoculation.

### 3.5 Bacterial colonization and persistence in mice

**Figure 3:**
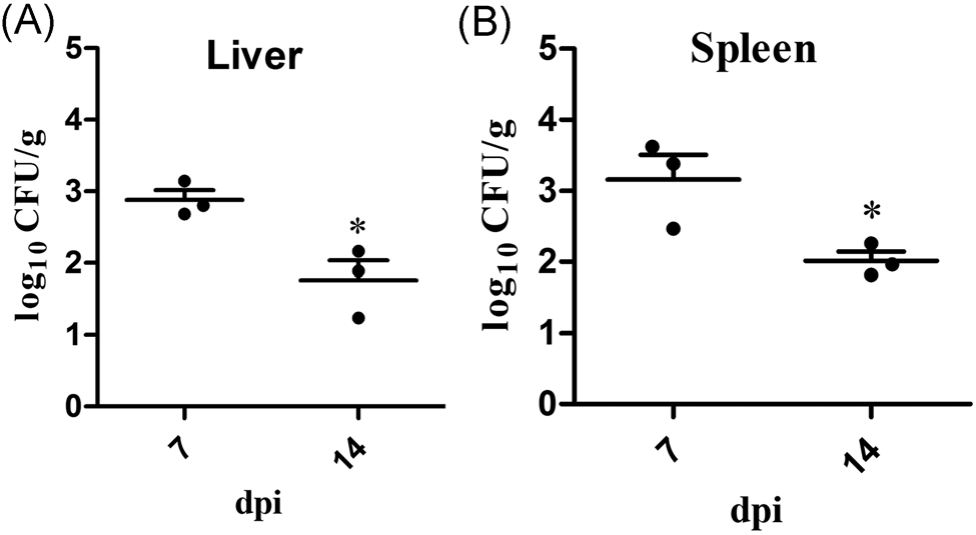
Bacterial recovery from the liver and spleen of the inoculated mice. Group 1 was intraperitoneally inoculated with 2 × 10^5^ CFU SPA017Δ*sptP* in BALB/c mice, and Group 2 only received 100 μl PBS. Values represent the mean ± *SEM* log_10_ CFU/g. All liver and spleen samples of Group 2 were negative. CFU, colony‐forming unit; dpi, days postinoculation; PBS, phosphate‐buffered saline. *p < .05

Bacteria were not isolated from spleen and liver samples of control mice. SPA017Δ*sptP* was isolated from the spleen and liver samples of the immunized mice at 7 and 14 dpi, the number of SPA017Δ*sptP* has significantly decreased from 7 to 14 dpi in both liver and spleen (*p* < .05), as shown in Figure 3.

### 3.6 Cellular and humoral immune responses

**Figure 4:**
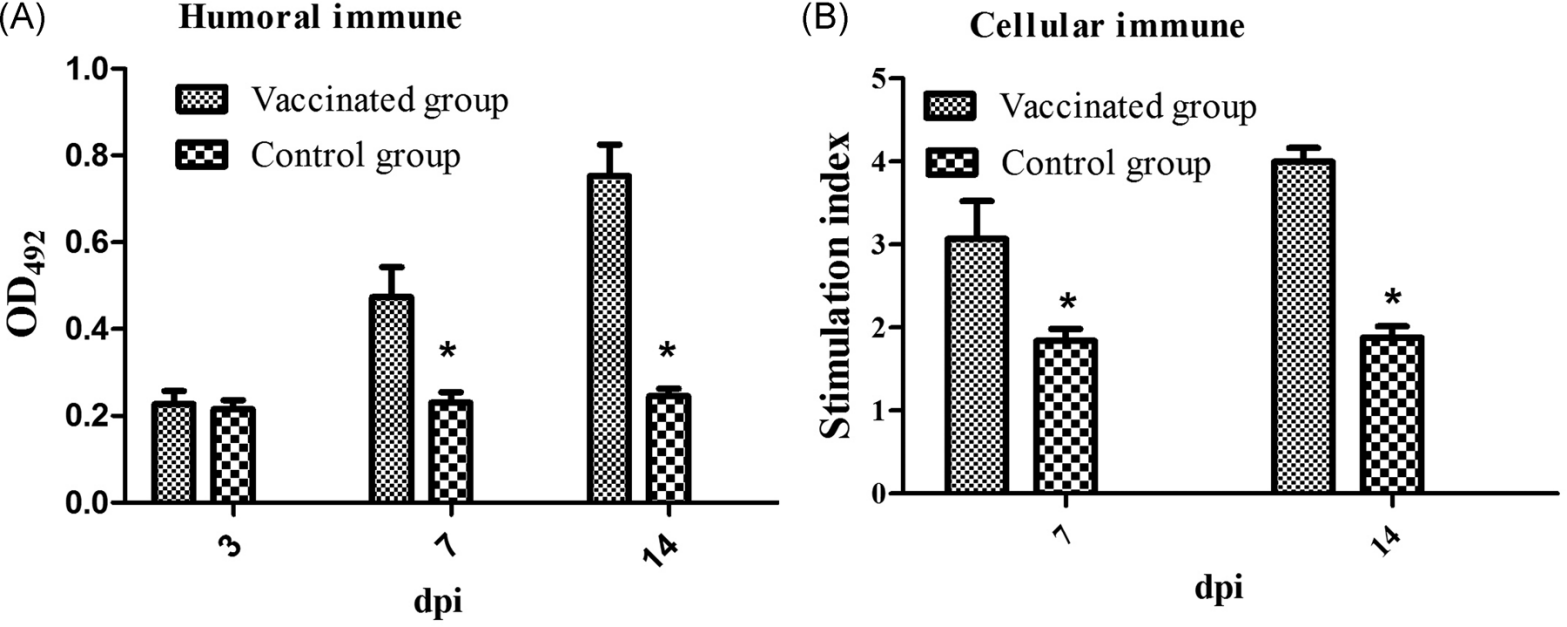
Humoral and cellular immune responses after vaccination. (A) Determination of serum IgG levels. (B) Stimulation index (SI) of mice lymphocyte samples. Groups 1 and 2 refer to Figure 3. Values represent the mean ± *SEM*. *Significant difference compared to Group 2, *p* < .05. dpi, days postinoculation; IgG, immunoglobulin G; OD, optical density

Cellular and humoral immune responses were measured after intraperitoneal inoculation with SPA017Δ*sptP*. The mice in Group 1 (inoculated with SPA017Δ*sptP*) showed significantly higher serum IgG levels and elevated SI values at 7 and 14 dpi compared to Group 2, as shown in Figure 4A,B (*p* < .05).

### 3.7 Evaluation of protection

The percent survival of mice intraperitoneally immunized with the mutant SPA017Δ*sptP*, followed by intraperitoneal challenge with the parent *Salmonella* Paratyphi A strain SPA017 at 14 days postimmunization was shown in Table 4. The survival rate of immunized mice (Group A) was 100%; all mice of Group B died following challenge with SPA017. Compared with mice in Group C, the immunized mice exhibited minor and brief clinical symptoms. Severe clinical symptoms, including anorexia, depression, diarrhea, morbidity, as well as high mortality, were observed in Group B. Effective protection was offered by SPA017Δ*sptP*.

**Table 4:** Protective rates of SPA017Δ*sptP* in mice via intraperitoneal vaccination

Group	Vaccination		Number	Challenge			Survivors/total	Survival rate (%)
	Strain	Dose (CFU)		Strain	Route	Dose (CFU)		
A	SPA017Δ*sptP*	2 × 10^5^	5	SPA017	Intraperitoneally	1 × 10^3^	5/5	100**
B	PBS	–	5	SPA017	Intraperitoneally	1 × 10^3^	0/5	0
C	PBS	–	5	PBS	Intraperitoneally	–	5/5	100

** *p* < .01 for comparison of Group A with Group B.

Abbreviations: CFU, colony‐forming unit; PBS, phosphate‐buffered saline.

## 4 DISCUSSION

In the present study, an *sptP* gene deletion mutant (SPA017Δ*sptP*) of *Salmonella* Paratyphi A SPA017 was constructed successfully, and then its characterization was analyzed based on growth, biochemical testing, adhesion and invasion assays, virulence assay. Finally, the protective efficacy of SPA017Δ*sptP* was assessed in mice.

We all know that the *sptP* gene was obtained through horizontal gene transfer with SPI1,^21^ so we suspect that SptP is not an essential metabolic factor for *Salmonella*. After the mutant SPA017Δ*sptP* of *Salmonella* Paratyphi A and its complementation CoSPA017Δ*sptP* were successfully constructed, growth curves and biochemical testing indicated that the mutant of *sptP* gene did not affect the growth and biochemical properties of *Salmonella* Paratyphi A. Also, the loss of *sptP* gene did not influence the growth of *Salmonella enteritidis*,^15^ this result was consistent with that of us.

Adhesion is essential and initial for *Salmonella* infection; invasion is the second step following adhesion, and then results in diseases.^21^ Our results showed that loss of *sptP* did not affect the ability of adhesion of *Salmonella* Paratyphi A in RAW264.7 and Caco‐2 BBE, but lack of *sptP* gene can decrease the invasive ability of *Salmonella* Paratyphi A into these two cell lines significantly. Similar results were also observed after the loss of *sptP* in *Salmonella enteritidis* and *Salmonella typhimurium*.^11^, ^15^ However, a few studies found that lack of *sptP* from *S. typhi* did not affect the invasive efficiency into HeLa cells due to the transcriptional regulator, TviA, which can be found in only *Salmonella typhi* and repress T3SS1 and flagella in *Salmonella typhi*.^11^, ^20^, ^21^, ^22^, ^23^ To determine the influence of the *sptP* on virulence in *Salmonella* Paratyphi A in mice, the LD_50_ of the mutant SPA017Δ*sptP* was measured. Our result demonstrated that loss of *sptP* can significantly reduce the virulence of *Salmonella* Paratyphi A; it was consistent with previous studies.^11^, ^15^

Vaccination is an ideal choice to control and prevent *Salmonella* infection.^24^ Finally, we measured the protective efficacy of SPA017Δ*sptP* in mice after intraperitoneal vaccination based on changes in body weight and clinical symptoms, SPA017Δ*sptP* colonization and persistence, cellular and humoral immune responses, as well as protective rates. Our results show that SPA017Δ*sptP* had no influence on the growth of the inoculated mice and did not cause any adverse reactions in mice, which is an important indicator for evaluating the safety of vaccines. The mutant SPA017Δ*sptP* can persist at least 14 days in immunized mice. It provided the condition for SPA017Δ*sptP* to induce the immune responses. Induction of effective cellular and humoral immune responses is an important indicator of the *Salmonella* vaccine.^24^ Our results showed that the mutant SPA017ΔsptP considerably induced specific cellular and humoral immune responses in mice. Finally, to evaluate the protective efficiency of SPA017Δ*sptP* in mice, the parental virulent strain SPA017 was used for the challenge. Our results demonstrated that the mutant SPA017ΔsptP offered efficient protection against the infection of *Salmonella* Paratyphi A. A previous study had shown that the loss of *sptP* from *Salmonella enteritidis* could also provide efficient protection in BABL/c mice.^15^ Also, based on the mutant SPI2, yncD and/or htrA from *Salmonella* Paratyphi A also showed the potential of being a live‐attenuated vaccine candidate.^16^, ^25^, ^26^ Next, further trials are needed for us to evaluate in detail the protective efficacy of the mutant SPA017Δ*sptP* based on other aspects, such as the doses, and pathway of vaccination, and so on.

Overall, our research shows that the *sptP* mutant (SPA017Δ*sptP*) of *Salmonella* Paratyphi A is attenuated, immunogenic, and protective in mice, and SPA017ΔsptP has the potential to become a live‐attenuated vaccine.

## CONFLICT OF INTERESTS

The authors declare that there are no conflict of interests.

## AUTHOR CONTRIBUTIONS

Qunli He and Junlei Yin designed the experiments; Pengtao Pan, Qunli He, and Junlei Yin conducted the experiments; Pengtao Pan, Fanyu Zou, and Chuanshan He performed the experiments; Pengtao Pan, Fanyu Zou, Chuanshan He, and Junlei Yin analyzed the data and drafted the manuscript; Qunli He and Junlei Yin finalized the manuscript.

## Data Availability

The data that support the findings of this study are available from the corresponding author upon reasonable request.
